# A retrospective chart review assessing antibiotic treatment of hospitalized patients with discordant *Clostridioides difficile* assays in an urban hospitalized setting

**DOI:** 10.1017/ash.2024.60

**Published:** 2024-04-23

**Authors:** Clare Stoddart, Irene Kuo, Matthew A. Spence, Tara N. Palmore

**Affiliations:** 1 The George Washington University Milken Institute School of Public Health, Washington, DC, USA; 2 Department of Epidemiology, The George Washington University Milken Institute School of Public Health, Washington, DC, USA; 3 The George Washington University Hospital, Washington, DC, USA; 4 The George Washington University School of Medicine & Health Sciences, Washington, DC, USA

## Abstract

*Clostridioides difficile* infection (CDI) threatens vulnerable populations in health care. Two-step testing improves specificity, avoiding over-treatment. This study analyzed inpatient records to estimate diagnostic outcomes and identify characteristics associated with treatment after discordant testing. Among discordant patients, those aged 65+ years were significantly more likely to be prescribed antibiotics (67% vs 39%).

## Background

*Clostridiodes difficile* is a gram-positive, toxigenic anaerobic bacillus which can colonize the diverse human colonic microbiome or cause invasive infection.^1^ The diagnostic tests utilized by hospitals to detect *C. difficile* include enzyme immunoassays (EIAs) to detect *C. difficile* glutamate dehydrogenase antigen and/or toxin and nucleic acid amplification tests (NAAT) such as polymerase chain reaction (PCR) tests which detect toxin-B genes. Current guidelines recommend a multistep algorithm for EIA testing with PCR to strengthen sensitivity and clinical evidence in diagnosing *Clostridioides difficile* infection (CDI).^2^ Interpreting only one positive result is considered insufficient for stand-alone CDI diagnosis where testing algorithms have been implemented. Discordant algorithm results, where the EIA and PCR results for toxin presence disagree, could indicate *C. difficile* colonization, which is not typically treated, or an alternative diagnosis. Treatment decisions can be influenced by patient and clinician factors. A recent meta-analysis investigating clinical outcomes for EIA-positive and NAAT-negative patients emphasized the need for additional data on discordant patients to inform antibiotic prescription decision-making.^3^

We conducted an inpatient chart review at an urban, academic tertiary hospital to assess the use of the multistep testing algorithm to evaluate invasive CDI and treatment decisions. The algorithm instructs a dual antigen and toxin EIA to be performed only following a positive result from a PCR. Practitioners are recommended to treat for CDI if a patient’s PCR and both EIA test components are positive. However, the proportion of patients with discordant results who are treated for CDI is not well documented, nor is the association of demographic factors with treatment. Demographically, the incidence of healthcare-associated CDI per 100,000 is greater in females, individuals aged 65+ years, and non-White people.^4^ We aimed to investigate if age, sex, and race or ethnicity are associated with CDI treatment in patients with discordant test results at an urban academic hospital over a 5-year period.

## Methods

**Figure 1.**
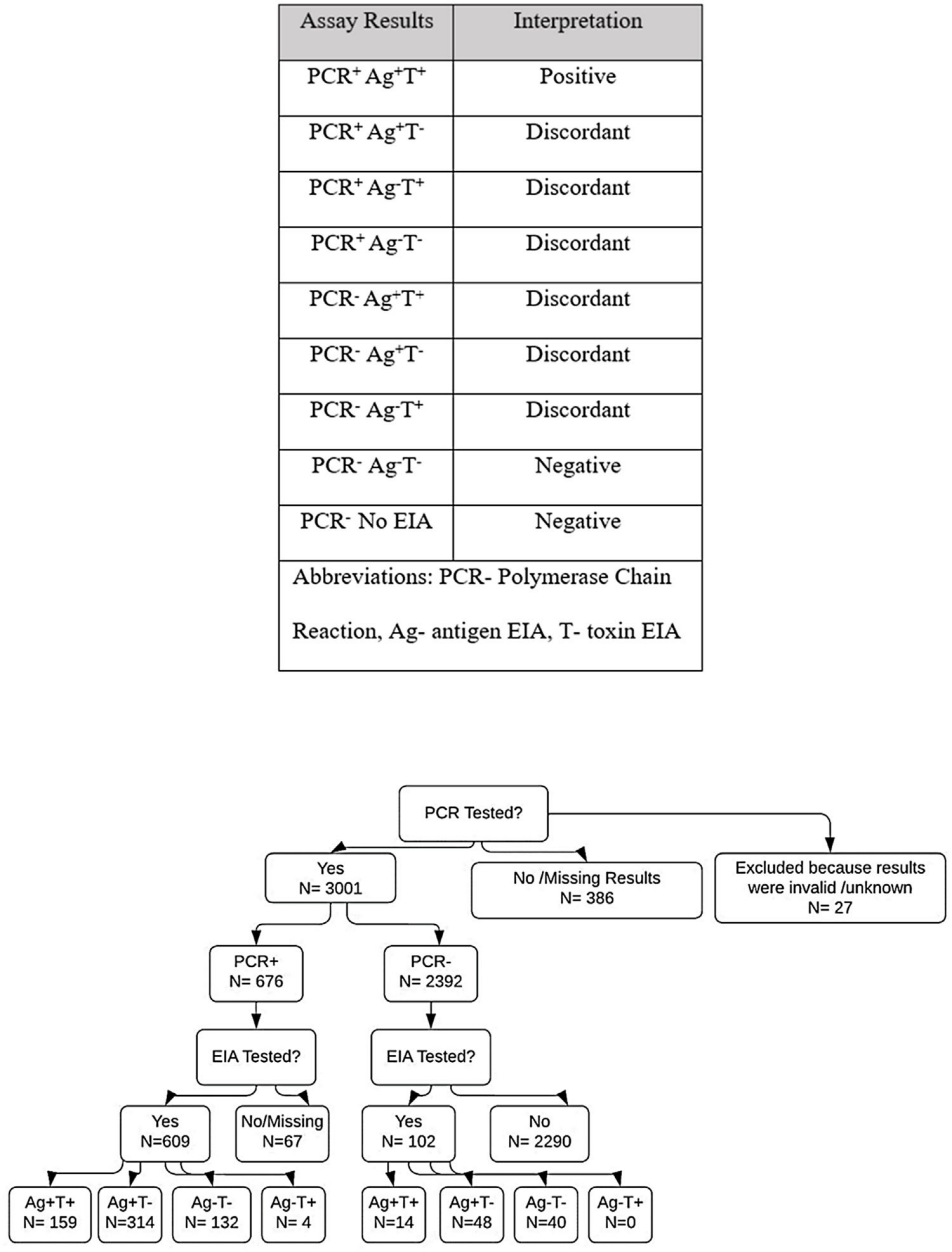
Algorithm assay results and their corresponding clinical interpretation used in this study. Flow chart visualizing the testing categories and group size for which the distribution of antibiotic treatment was then calculated (*N* = 3414).

We reviewed records of patients admitted to the urban academic hospital (George Washington University Hospital) and tested for CDI from January 1, 2018, to December 31, 2022 to assess testing algorithm adherence, results, and treatment of CDI.^5,6^ The multistep testing algorithm to diagnose invasive CDI was first implemented at this hospital in 2017. Each component—antigen or toxin within EIA, and PCR—can produce a positive or negative result indicating substrate presence in the sample. Interpretations of test results into CDI diagnostic categories are listed in Figure 1.

Hospital pharmacy records were used to categorize each patient as treated or untreated based on their receipt of oral vancomycin or fidaxomicin, and metronidazole (utilized only as an adjunctive therapy to vancomycin). Those with no pharmaceutical data indicating vancomycin or fidaxomicin prescription were considered untreated.

We computed descriptive statistics based on testing category and treatment outcome in the matched dataset after de-identification, along with the demographic (age, sex, race, and ethnicity) variables contained in the discordant group (Table 1). We examined the distribution of patients in each testing category and computed the percent with the antibiotic treatment outcome to test our hypothesis. We used χ^2^ testing to assess for association in the discordant category by previously listed correlates and used multiple-logistic regression to identify independent associations of antibiotic treatment. All analyses were conducted using SAS (v9.4, Cary, NC).

**Table 1.** Characteristics of treated versus untreated patients in the discordant testing category (*N* = 512)

	Total, N	Treated	Untreated	Odds ratio [95% CI]	*P*-value
Age (years)					
*0–65*	318	90	228	1.0 [ref.]	
*65+*	194	78	116	1.70 [1.17, 2.48]	0.0054*
Sex					
*Male*	222	73	150	1.0 [ref.]	
*Female*	290	96	194	1.03 [0.71, 1.50]	0.8727
Race					
*Amer. Indian/Alaska Native*	0	0	0	—	—
*Asian*	5	1	4	0.89 [0.17, 4.68]	0.8941
*Black/African American*	287	79	208	1.0 [ref.]	—
*Multiple*	6	2	4	1.12 [0.20, 6.19]	0.8991
*White*	94	36	58	1.49 [0.95, 2.37]	0.0827
*Unknown*	27	7	20	0.971 [0.45, 2.11]	0.9415
Ethnicity					
*Non-Hispanic/Latino*	394	120	274	1.0 [ref.]	—
*Hispanic/Latino*	10	2	8	0.741 [0.23, 2.37]	0.6131
*Unknown*	17	4	13	1.165 [0.48, 2.84]	0.7367

Reference groups: age = 0–65, sex = male, race = Black/African American, ethnicity = non-Hispanic Latino. Abbreviations: CI = confidence interval, * = *P* < 0.05.

## Results

A total of 3414 patients were included in the analysis, each of whom was tested with some part of the algorithm [Figure 1]. Among them, 413 patients (12.1%) in the data set were missing either PCR results or EIA results, or their results were listed non-applicable and were therefore excluded from analysis. The remaining 3001 patients were initially tested for CDI using a PCR test; of those, 22.5% were positive; 609 (90.1%) of PCR-positive patients were further tested using the *C. difficile* antigen/toxin EIA algorithm. Overall, 5.3% (159/3001) of patients tested positive across all algorithm components.

Of the negative group (PCR^-^ with no EIA or Ag-/T-), 183/2330 (7.85%) were treated. In the discordant category, which included a range of testing outcome combinations with both positive and negative PCR, 168 patients (32.81%) were treated with antibiotics. Among those with both positive PCR, antigen and toxin EIA, 134 (84.28%) patients were treated.

Of patients with both test components on record (regardless of algorithm guidelines), 512 (72.0%) had discordant PCR and EIA results. Among this group, age 65+ years was found to be significantly associated with receiving antibiotic treatment (aOR = 1.67; 95% CI [1.13, 2.45], *P* = .0094), after adjusting for sex, race, and ethnicity.

Patients with missing PCR but an existing EIA result or positive PCR with a missing EIA result may have undergone the missing tests, but non-adherence to the algorithm or failure in record-keeping have left gaps in the data. Among those with missing PCR results but existing positive EIA results, 47.34% received treatment and 52.66% did not receive treatment. Of the 67 patients with a positive PCR but no recorded EIA, 49 (73.13%) were treated.

## Discussion

In this single-center study, the pattern of CDI treatment among testing groups is consistent with existing literature in settings using a two-step algorithm.^7^ A Veterans Affairs study reviewing patients with positive PCR and negative toxin EIA found 29.5% of discordant patients were treated.^8^ Fewer than 10% of those categorized in our facility as CDI-negative and >80% of those categorized as CDI-positive were treated with antibiotics, suggesting relatively adherent stewardship practices consistent with the recommended algorithm.

CDI risk is estimated to increase by 2% every year over age 65 years and can cause severe complications in patients of advanced age.^9^ These data may explain the finding that patients aged 65+ years with discordant test results had nearly twice the odds of receiving CDI treatment compared to those under 65 years of age. Clinicians may have been concerned about delaying intervention for older patients with possible CDI despite the discordant or incomplete test results. No other demographic characteristics were associated with treatment, suggesting age-associated comorbidities contributed to the decision to prescribe discordant patients CDI treatment. It is also possible that prior antibiotic use or hospitalizations may explain treatment in the double negative and discordant testing category,^10^ but these variables were lacking for this analysis.

Limitations of this study included the considerable proportion of missing PCR and EIA testing data, which could bias our findings. These results were not found in patient charts or microbiology lab logs, suggesting these tests were either not performed, results were not reported or retrievable from hospital records, or results were written as “non-applicable” and could not be interpreted as positive or negative. Another limitation includes the scarcity of additional covariates available for analysis, which may have resulted in residual confounding. This analysis also does not include patients who were not tested for CDI but still treated or treated outside the hospital. The retrospective study took place in a single urban tertiary care center, and results may not be generalizable to other facilities. Because facilities use a variety of diagnostic algorithms, the classification of discordance used in this study may not be broadly applicable.

*C. difficile* is an increasingly burdensome problem in healthcare settings and continues to cause significant morbidity and mortality. Diagnostic stewardship algorithms and guidelines are designed to reduce treatment of patients who have *C. difficile* colonization rather than CDI. We plan to leverage our findings to understand more about patients with discordant test results and continue the effort to reduce the burden of nosocomial CDI while prioritizing diagnostic stewardship.

## Supporting information

Stoddart et al. supplementary material Stoddart et al. supplementary material

## Supplementary material

For supplementary material accompanying this paper visit http://doi.org/10.1017/ash.2024.60. click here to view supplementary material

## Manuscript preparation

Only listed authors provided assistance in preparing and editing the manuscript.
