# Supplementary material for: A retrospective chart review assessing antibiotic treatment of hospitalized patients with discordant Clostridioides difficile assays in an urban hospitalized setting
Source: Antimicrob Steward Healthc Epidemiol. 2024 Apr 23;4(1):e57. doi: 10.1017/ash.2024.60 (PMC11036419; doi:10.1017/ash.2024.60)
Supplement: Stoddart et al. supplementary material [file S2732494X24000603sup001.docx]

**Supplementary Material**

| Table S1: Characteristics of All Subjects Tested For CDI Using Two-Step Algorithm From 2018-2022 | | | | | |
| --- | --- | --- | --- | --- | --- |
| Demographic Characteristic | Total  (N=3414)  [col. %] | Negative  N=  [row %] | Discordant  N=  [row %] | Positive  N=  [row %] | Not Included  N=  [row %] |
| Age |  |  |  |  |  |
| *<65* | 2060  [60.34] | 1433  [61.50] | 318  [62.11] | 80  [50.31] | 229  [55.45] |
| *≥65* | 1354  [39.66] | 897  [38.50] | 194  [37.89] | 79  [49.69] | 184  [44.55] |
| Sex |  |  |  |  |  |
| *Male* | 1671  [48.97] | 1178  [50.58] | 222  [43.36] | 82  [51.57] | 189  [45.87] |
| *Female* | 1743  [51.03] | 1151  [49.42] | 290  [56.64] | 77  [48.43] | 223  [54.13] |
| Race |  |  |  |  |  |
| *Amer. Indian/ Alaska Native* | 1  [0.03] | 0  [0] | 0  [0] | 0  [0] | 1  [0.24] |
| *Asian* | 50  [1.47] | 36  [1.55] | 7  [1.37] | 2  [1.26] | 5  [1.22] |
| *Black Or African American* | 2143  [62.84] | 1432  [61.48] | 359  [70.12] | 98  [61.64] | 254  [61.95] |
| *Multiple* | 39  [1.14] | 28  [1.20] | 6  [1.17] | 3  [1.89] | 2  [0.49] |
| *White* | 846  [24.81] | 594  [25.50] | 105  [20.51] | 37  [23.27] | 110  [26.83] |
| *Unknown* | 331  [9.71] | 239  [10.26] | 35  [6.83] | 19  [11.95] | 38  [9.27] |
| Ethnicity |  |  |  |  |  |
| *Non-Hispanic /Latino* | 3048  [89.34] | 2060  [88.45] | 474  [92.58] | 137  [86.16] | 377  [91.73] |
| *Hispanic/ Latino* | 162  [4.75] | 131  [5.62] | 15  [2.93] | 9  [5.66] | 7  [1.70] |
| *Unknown* | 198  [5.81] | 138  [5.92] | 23  [4.50] | 13  [8.18] | 24  [5.84] |
| Missing Attribute Values: Sex N=2, Race N=4, Ethnicity N=3. | | | | | |

| **Table S2: Distribution of Antibiotic Treatment Outcome (N=3414)** | | | |
| --- | --- | --- | --- |
|  | Total  N [col %] | Treated  [row %] | Untreated  [row %] |
| Negative | 2330  [68.25] | 183  [7.85] | 2147  [92.15] |
| PCR**^-^**  PCR**^-^**Ag**^-^**T**^-^** |  |  |  |
| Discordant | All  512 [15.00]  *PCR^+^ Disc.*  450  *PCR^-^ Disc.*  62 | All  168 [32.81]  *PCR^+^ Disc.*  127  *PCR^-^ Disc*  41 | All  344 [67.19]  *PCR^+^ Disc.*  323  *PCR^-^ Disc*  21 |
| PCR**^+^** Ag**^+^**T**^-^**  PCR**^+^** Ag**^-^**T**^+^**  PCR**^+^** Ag**^-^**T**^-^**  PCR^-^ Ag^+^T^+^  PCR**^-^** Ag**^+^**T**^-^**  PCR**^-^** Ag**^-^**T**^+^** |  |  |  |
| Positive | 159  [4.66] | 134  [84.28] | 25  [15.72] |
| PCR**^+^** Ag**^+^**T**^+^** |  |  |  |
| Missing PCR or Ag/T Test Result | 386  [11.31] | 200  [51.81] | 186  [48.19] |
| Other/Unknown | 27  [0.78] |  |  |
| Abbreviations: PCR=Polymerase Chain Reaction, Ag= Antigen, T=Toxin,  %=percent of that testing category | | | |
